# Workers’ experiences with compensated sick leave due to musculoskeletal disorder: a qualitative study

**DOI:** 10.1186/s40557-014-0033-0

**Published:** 2014-11-04

**Authors:** Min Choi, Hyoung-Ryoul Kim, Jinwoo Lee, Hye-Eun Lee, Junsu Byun, Jong Uk Won

**Affiliations:** 1 Department of Occupational and Environmental Medicine, College of Medicine, the Catholic University of Korea, 222 Banpo-Daero, Seocho-Gu, Seoul 137-701, Republic of Korea; 2 Research Institute for Alternative Worker’s Movements, 14-1 Donggyo-ro 29, Mapo-Gu, Seoul 121-865, Republic of Korea; 3 Department of Preventive Medicine, College of Medicine, Yonsei University, 50-1 Yonsei-ro Seodaemun-gu, Seoul 120-752, Republic of Korea

**Keywords:** Musculoskeletal disease, Workers’ compensation, Occupational injuries, Qualitative research

## Abstract

**Objectives:**

The most common occupational disease that is compensated by Industrial Accident Compensation Insurance (IACI) in Korea is musculoskeletal disease (MSD). Although complaints about the workers’ compensation system have been raised by injured workers with MSD, studies that examine workers’ experiences with the Korean system are rare. This paper is a qualitative study designed to examine injured workers’ experiences with the workers’ compensation system in Korea. The aim of this study is to explore the drawbacks of the workers’ compensation system and to suggest ways to improve this system.

**Methods:**

All workers from an automobile parts factory in Anseong, GyeongGi province who were compensated for MSD by IACI from January 2003 to August 2013 were invited to participate. Among these 153 workers, 142 workers completed the study. Semi-structured open-ended interviews and questionnaires were administered by occupational physicians. The responses of 131 workers were analyzed after excluding 11 workers, 7 of whom provided incomplete answers and 4 of whom were compensated by accidental injury. Based on their age, disease, department of employment, and compensation time, 16 of these 131 workers were invited to participate in an individual in-depth interview. In-depth interviews were conducted by one of 3 occupational physicians until the interview contents were saturated.

**Results:**

Injured workers with MSD reported that the workers’ compensation system was intimidating. These workers suffered more emotional distress than physical illness due to the workers’ compensation system. Injured workers reported that they were treated inadequately and remained isolated for most of the recuperation period. The compensation period was terminated without ample guidance or a plan for an appropriate rehabilitation process.

**Conclusions:**

Interventions to alleviate the negative experiences of injured workers, including quality control of the medical care institutions and provisions for mental and psychological care for injured workers, are needed to help injured workers return to work earlier and more healthy.

## Introduction

Huge movements led by the manufacturing trade union in South Korea in the early 2000s have highlighted the struggles associated with workers’ compensation for work-related musculoskeletal disorders (WRMSD). Public awareness for WRMSD was raised as a result of these movements, and the number of claims for musculoskeletal disorders (MSD) filed with Industrial Accident Compensation Insurance (IACI) was increased. MSD is now the most common occupational disease compensated by IACI in Korea [1]. However, the approval rates for WRMSD have been decreasing over the past several years. For example, the approval rate for WRMSD was 47.7% in 2010 compared with 55.3% in 2007 [2].

Injured workers with MSD seldom recover completely, and their symptoms persist for a long duration in many cases. The results from a survey conducted in Korea in 2011 found that 44.2% of injured workers with a lumbar herniated intervertebral disc (HIVD) who reached the end of their compensation period reported that their severe symptoms persisted even after their return to work [3]. However, it is difficult to extend the duration of medical care benefits under the current system.

Although several complaints about the workers’ compensation system in Korea have been raised by injured workers with MSD, studies that examine workers’ experiences with this system are rare. Lee et al. measured workers’ satisfaction with the workers’ compensation system, but this study was limited to workers who were injured by trauma [4]. In a satisfaction research survey conducted by Korea Workers’ Compensation and Welfare Service (KCOMWEL), 71.8% of respondents answered that they were satisfied with the workers’ compensation system. However, this study was limited to injured workers who were covered by the case management service [5]. There are few studies on satisfaction with the workers’ compensation system among injured workers with MSD.

Most studies about WRMSD in Korea were designed to investigate the symptom prevalence rate and work-related risk factors in diverse occupations and industries using ergonomic evaluations [6]–[8]. In addition, some studies have examined the structural aspects of WRMSD management [9] and the conditions of injured workers with MSD [2],[3]. However, it is hard to find the voices and detailed experiences of injured workers in these studies.

It is difficult to derive a implication that can be used to improve the system from the responses of affected workers’ experiences in quantitative studies. These studies are usually based on variables gathered from published epidemiological investigations and focus on the number of affected workers, the amount of compensation payment, and the approval rates. Whereas, in a qualitative study of 400 workers’ compensation cases in the United States, the majority of injured workers reported that the workers’ compensation system was cumbersome, dissatisfactory, and insulting [10].

To overcome the lack of research on workers’ experiences with the workers’ compensation system, this paper presents a qualitative study that used in-depth interviews to explore the drawbacks of the workers’ compensation system and to generate suggestions to improve this system in Korea.

## Materials and methods

### Participants

All workers from an automobile parts factory located in Anseong, GyeongGi province who were compensated for MSD by IACI from January 2003 to August 2013 were invited to participate in the study. In this factory, labor union was vitalized from 2002. In 2003, 21 workers of this union applied workers’ compensation for WRMSD as a group for the first time. After struggles going along with national movement, all of them were compensated by IACI. From that time, around 90 workers were compensated for WRMSD for about 1 year. Among about 500 workers (including office workers) hired in this factory, 153 workers has been compensated for MSD for the last 10 years.

Among these 153 workers, 142 workers completed the interview, yielding a response rate of 92.8%. Semi-structured open-ended interviews and questionnaires were administered by occupational physicians between August 20 2013 and September 4 2013. Eleven workers were excluded from the analysis: seven workers provided incomplete answers and four workers were compensated by accidental injury. The responses of 131 injured workers were included in the final analysis.

Based on their age, disease, department of employment, and compensation time, 16 of the 131 workers were invited to participate in an individual in-depth interview. In-depth interviews were conducted by one of three occupational physicians until the interview contents were saturated.

### Data collection

#### Questionnaires

Before the interview, each worker completed self-administered questionnaires. These questionnaires included questions about the compensation period, the time to obtain approval, the degree of satisfaction with the compensated medical care and compensation services, depressive symptoms during the compensation period, family relationships during the compensation period, and work position transition and relationship changes with co-workers following their return to work.

#### In-depth interview

Semi-structured and open-ended individual interviews were conducted between August and September 2013. The individual interviews focused on the compensation system, the medical treatment compensated by IACI, the process by which workers received rehabilitation, the process by which workers returned to work, psychosocial health status, and the workers’ experiences with the workers’ compensation system.

Open-ended questions were used to eliminate the interviewer’s preconceptions and to encourage participants to express their experiences in their own words. Some examples of interview questions are here.

 How was the applying and approval for workers compensation process?

 In your compensation periods, what was medical treatment?

 In your convalescence, what was the relationship with KCOMWEL?

 How was your family relationship and situation during convalescence?

 How did you feel during compensation periods?

 How was the return-to-work process?

 If you had experienced convalescence more than twice, what was the difference between one and another convalescence?

A semi-structured interview guide was used to ensure consistency between the 3 interviewers. The questions in the interview guide were developed by the 3 interviewers during 3 prior discussions about the qualitative methods to be used in the study. Each interview took 40 to 60 minutes to complete. All the in-depth interviews were audio recorded and transcribed.

### Data analysis

A frequency analysis was conducted for each questionnaire item. Notes from the interviewers and verbatim transcriptions were collected. Researchers read through these materials repeatedly and selected meaningful phrases or sentences that were directly related to the workers’ experiences with the compensation system. These phrases and sentences were coded and categorized by themes. Subcategories were derived from those themes, and these subcategories were classified into categories representing the workers’ experiences with workers’ compensation for MSD.

Peer-review discussion was implemented to attain validity of the qualitative methods. Detailed descriptions of the participants and the circumstances surrounding the interviews were presented to accomplish transferability [11].

This project received ethical approval from the Institutional Review Board of the Catholic University of Korea. All the participants volunteered to participate in the study and signed informed consent forms.

## Results

### Survey results

The 131 study subjects consisted of 129 males (98.5%) and 2 females. The average age of all the respondents was 48.1 years (S.D = 5.74). Compensation was approved for the first time in 2003 for 77 of the respondents (59.2%), and in 2004 for 22 of the respondents (16.9%). Only 23.8% of the respondents received approval for compensation for the first time after 2005. The neck and shoulders were the most common injury sites among respondents (53.4%), followed by back (43.5%).

The median duration of compensation was 7.0 months. The compensation duration was less than 3 months for 17.8% of the respondents and more than 12 months for 12.4% of the respondents. Ninety-six respondents (73.3%) extended the duration of their compensation and 22 respondents (16.9%) were re-compensated for the same disease. Table 1 outlines the demographic and compensation-related characteristics of the respondents.

**Table 1:** General characteristics of questionnaire responders (including in-depth interviewee) (n = 131)

**Variables**		**n**	**(%)**
Gender	Male	129	(98.5)
	Female	2	(1.5)
Age (years) (n = 127)	Mean (S.D*) 48.1 (5.74)		
	≤ 39	8	(6.3)
	40-49	69	(54.3)
	50-58	50	(39.4)
Initial year of compensation approval (n = 130)			
	2003	77	(59.2)
	2004	22	(16.9)
	2005	9	(6.9)
	2006	4	(3.1)
	2007	9	(6.9)
	2008	3	(2.3)
	2009	2	(1.5)
	2012	4	(3.1)
Injury site (n = 137)	Neck, Shoulder	70	(53.4)
	Arm, wrist, hand	8	(6.1)
	Back	57	(43.5)
	Knee, Leg	2	(1.5)
Compensation duration (month) (n = 129)	Median 7.0		
	less than 3 months	23	(17.8)
	less than 6 months	36	(27.9)
	less than 12 months	54	(41.9)
	more than 12 months	16	(12.4)
Extension of compensation duration			
	No	35	(26.7)
	Once	49	(37.4)
	More than twice	47	(35.9)
Re-compensation for same disease (n = 130)			
	No	108	(83.1)
	Yes	22	(16.9)
Medical operation			
	No	106	(80.9)
	Yes	25	(19.1)

Note: Numbers do not always add up to 131 because not all individuals reported each characteristic. Workers could respond more than 1 injury site.

^*^S.D Standard deviation.

Ninety-one respondents (69.5%) received appropriate treatment for their disease before their compensation was approved. However, 4.6% of the respondents did not start medical treatment until after their compensation was approved.

Among all the respondents, 76 (58.0%) reported that their physician asked about their work duties and the posture that is required during their work. However, only 43 respondents (34.1%) consulted their physicians about their return to work. The majority of respondents (78.6%) underwent medical treatment that was not covered by workers’ compensation (Table 2).

**Table 2:** Treatment-related characteristics of subjects (n = 131)

**Variables**		**n**	**(%)**
Treatment before approval recognition	Appropriate	91	(69.5)
	Inappropriate	34	(26.0)
	No treatment before approval	6	(4.6)
Discussion about work with physician	No	55	(42.0)
	Yes	76	(58.0)
Consult from physician about return to work (n = 126)	No	83	(65.9)
	Yes	43	(34.1)
Satisfaction with medical treatment (n = 130)	Very satisfied	3	(2.3)
	Mostly satisfied	75	(57.7)
	Mostly unsatisfied	46	(35.4)
	Very unsatisfied	6	(4.6)
Treatment not covered by workers’ compensation (n = 185)	None	28	(21.4)
	Exercise	54	(41.2)
	Alternative medicine	51	(38.9)
	Dietary supplement	40	(30.5)
	Others	12	(9.2)

Note: Numbers do not always add up to 131 because not all individuals reported each characteristic. Workers could respond more than 1 treatment not covered by workers’ compensation.

### In-depth interview results

#### General characteristics of in-depth interviewees

All 16 participants who completed the in-depth interview were males aged 40 to 54 years. Their compensation duration ranged from 6 to 48 months. Six participants were re-compensated for the same disease. Seven participants returned to the same position after their compensated sick leave. Ten participants were compensated for back problems, such as a lumbar herniated intervertebral disc (HIVD), and six participants were compensated for neck and shoulder diseases, such as myofascial pain syndrome (Table 3).

**Table 3:** Characteristics of in-depth interview subjects (n = 16)

**Subject**	**Age**	**Year of the initial compensation**	**Disease**	**Compensation duration (month)**	**Year of the second compensation**	**Return to the same work**
A	52	2003	HIVD^*^, Lumbar	6	2006^†^	Yes
B	54	2003	HIVD, Lumbar	6	-	Yes
C	49	2003	HIVD, Lumbar	10	-	Yes
D	41	2003	HIVD, Lumbar	6	2010	
E	44	2003	HIVD, Lumbar	48	2013^†^	Yes
F	52	2003	HIVD, Lumbar	11	-	
G	53	2003	HIVD, Lumbar	12	2005	
H	48	2003	HIVD, Lumbar	6	2008^†^	Yes
I	52	2003	Myofascial pain syndrome	5	2009^†^	Yes
J	51	2003	Rotator cuff tear	8	2011^†^	
K	45	2003	HIVD, Cervical	10	2008^†^	
L	48	2003	HIVD, Cervical	6	-	Yes
M	40	2004	HIVD, Lumbar and cervical, Impingement syndrome	11	-	
N	41	2005	HIVD, Cervical, Myofascial pain syndrome	8	2007^†^	
O	49	2005	Lumbar strain	6	-	
P	46	2005	Lumbar strain	5	-	

^*^HIVD herniated intervertebral disc.

^†^The year of the second workers compensation, but compensated for different disease from the first compensated disease.

#### Categories of workers’ experiences with workers’ compensation for MSD

The in-depth interview participants’ experiences with workers’ compensation for MSD were classified into four categories: workers’ compensation system, medical services, psychological and emotional aspects, and the termination of compensation and return to work.

The participants who completed the in-depth interviews reported that the workers’ compensation system was intimidating and that they received negligible medical treatment. Under these circumstances, injured workers suffered more emotional distress than physical illness, and ended the compensation period and returned to work without feeling fully recovered. The 14 subcategories and 4 categories of workers’ experiences with workers’ compensation for MSD are shown in Table 4.

**Table 4:** Categories of workers’ experiences with workers’ compensation for musculoskeletal disease

**Subcategories**	**Categories**
Approval rate for musculoskeletal disease is low; therefore, claims for workers’ compensation are difficult.	Intimidating aspects of the workers’ compensation system
Approval decision is not based on fair work-relatedness evaluation.	
Injured workers need to strive for approval.	
Recuperation period and termination is determined without considering the state of the individual patient.	
Injured workers feel fear of stigma as a malingerer.	More emotional distress than physical illness
Injured workers are remained isolated most of the time during recuperation.	
Injured workers are anxious during the whole period (from approval to termination).	
It took 1 hour per day for medical treatment, and injured workers stayed at home alone for the rest of the day.	Poor treatment and neglect of injured workers
Physical therapy is the only treatment and there is almost no exercise therapy.	
Injures workers pursue other self-remedies due to suspicious therapeutic effect of compensated medical care.	
Medical judgment is not taken into consideration in the decision to extend or terminate the compensation duration.	
Recuperation period is ended by KCOMWEL*, injured worker in inadequate condition to return to work.	Difficult termination and return to work
There is no workplace-based rehabilitation and return to work program.	
Rules for work placement and transitions for returned workers are absent.	

^*^KCOMWEL Korea Workers’ Compensation and Welfare Service.

### Intimidating aspects of the workers’ compensation system

Many participants reported that the approval rate for compensation was low and that they experienced increased difficulty with gaining approval. Participants felt that KCOMWEL’s effort to shorten the compensated sick leave period was strengthening. Therefore, many participants reported that workers ‘gave up’ applying for workers’ compensation. As a result, participants perceived that workers’ claims for compensation had decreased and that self-paid treatments or company-paid treatments for work-related disease were increasing.

“Ah, nowadays everybody knows workers’ compensation approval is difficult. It’s hard. It’s just vain efforts applying to the workers’ compensation. They would send me and union staff on a fool’s errand. I thought that paying myself was more comfortable than getting compensation. Umm, the company would provide some money from a kind of collective insurance……. I judged it would not be approved in my case. It’s very hard to get approved”. (Participant D)

Some respondents thought that the work-relatedness of the case did not influence the decision for approval. Participants felt that the severity of the disease, the disease diagnosis, the necessity of an operation, the labor union, and the affected worker’s effort were more important determinants of whether a case was approved.

“To make a phone call to KCOMWEL, to appeal for my suffering to the advisory medical doctors, and to give frequent visits to KCOMWEL ……. Those actions made my case approved. In the workers’ compensation decision-making process, you have to struggle to get approved”. (Participant J)

Some workers also expressed the inconvenience associated with determining the sick leave duration and termination. Participants felt that a fixed sick leave duration for every affected worker on the basis of the diagnosis was not fair because everyone’s condition is unique. Respondents complained to KCOMWEL and physicians about terminating sick leave without sufficient communication between the patient and physician. In one worker’s case, during a non-operative treatment for lumbar HIVD, a member of KCOMWEL told the injured worker to choose to either extend the sick leave duration by undergoing an operation or finish the sick leave. As a result, the application of a standardized sick leave duration prolonged the sick leave.

### More emotional distress than physical illness

Many injured workers emphasized that the emotional distress was so serious that they suffered from more emotional distress than physical illness during their compensation period. A stigma of injured workers as malingerers spread widely. Injured workers therefore limited their interactions with co-workers and lived in isolation.

Participants described the compensation period as ‘a prison without bars’ and pointed out ‘I could feel easier after returning to work.’

“At that time, I was really self-conscious. ‘What do other people talk about in whispers when they see me doing exercise in the gym?’ They certainly have a thought of that patient should be in a hospital. So I did exercise alone and secretly”. (Participant N)

However, many interview participants also stigmatized other injured workers. All the participants expressed that they were ‘real patients’ and suffered from a lack of sympathy from other co-workers, but some participants said there were malingerers among compensated workers. Other participants also thought that repeatedly injured workers had poor self-management.

Some participants revealed they were under strain while waiting for compensation approval and extension of the compensation period [12]. Participants also felt anxiety about the disease progression during treatment. Some injured workers felt sorry because they could not meet obligations to their family and co-workers while injured. When the compensation period ended, participants continued to experience considerable distress due to anxiety about their remaining symptoms, a feeling of uneasiness about returning to work, and pressure to recover quickly.

“I always felt stress during that period. I had not smoked cigarettes until then, but I started to smoke during the compensation period”. (Participant L)

### Poor treatment and neglect among injured workers

Injured workers began medical treatment under the stressful circumstances of waiting for compensation approval. However, the quality of the medical care offered by the medical clinics was low. For example, one treatment was hot pack therapy, which was offered by the physical therapy unit of the company. Injured workers spent only an hour per day on treatment. The lack of a systematic treatment program gave injured workers no choice but to spend the rest of the day in their own homes, which reinforced their anxiety and sense of isolation.

Participants indicated that counseling and treatment by physicians was limited during the compensation period. Most of the medical services compensated included physical therapy and medication. Medical consultation that considered the work and occupation of the injured worker was rare, and exercise therapy was seldom provided. These results are consistent with the survey results, in which only 17% of respondents answered that they were offered exercise therapy, whereas physical therapy and medication were offered to 96.2% and 95.4% of respondents, respectively.

“It didn’t take a long time to get medical treatment. It took about an hour a day to have physical therapy. There was no meeting with the physician. Mostly, I got only physical therapy. So, the rest of the time… I had to stay in the house during the rest of the day”. (Participant I)

Some injured workers mentioned that they felt medical treatment during the compensation period was ineffective, so they sought alternative means like exercise. Some participants therefore depended on services such as alternative medicine that were not reimbursed by IACI.

The lack of appropriate medical services was more problematic when the compensation period was terminated. Most participants said that the decision to return to work or extend the compensation period was made with financial managers in a clinic instead of physicians.

“When I asked to extend compensation, the doctor told me nothing. The doctor told me, ‘Get a financial manager’s permission’. In this way, extending the application was made by a financial manager not a medical doctor”. (Participant D)

### Difficult termination and return to work

Many injured workers felt they were not fully recovered when the compensation period ended. These workers stated that the decision was made without enough explanation to the injured worker. In many cases, injured workers were persuaded to finish the compensation period by KCOMWEL officers rather than medical doctors.

In addition, no systematic fitness for work evaluation or workplace-based rehabilitation program exists. Therefore, many workers suffered from remaining symptoms following their return to work. In our study, 30.6% of respondents reported that their present musculoskeletal symptoms were similar or worse than their initial symptoms. Only 17.6% of respondents answered that their symptoms were fully or almost resolved.

“Because I went into the assembly line right after a long break, I had a rough time. My back was so weakened that even coughing could make serious back pain. So, I wore a lumbar pad for several months”. (Participant F)

Due to the lack of workplace-based rehabilitation, work fitness evaluation, and work adjustment programs, there is a gap between the work capacity and expected role of the injured worker who returns to work following the compensated sick leave. This gap must be filled by the non-injured co-workers.

All the participants returned to their own company after the compensation period. However, the placement of the injured worker was an issue following their return. No standard about whether the worker should return to the same job or a new job exists. Furthermore, the injured worker was not allowed to take part in the decision about where to be placed. This situation therefore brought conflict and stress to the returning injured worker. Each worker had a different preference for returning to their previous job or changing jobs. However, the awkward situation that resulted from the lack of a reasonable and transparent placement rule was a common experience among returned workers. One participant summarized this experience by saying,

“If there was a standard, I wouldn’t have a complaint. But I thought the process was unfair. Someone returned to their own department, the others couldn’t return to the department they worked in before. I dislike this situation. I think all of us have to be treated equally”. (Participant O)

## Discussion

Workers compensated by IACI for MSD found the workers’ compensation system to be intimidating. Their emotional distress, including stigmatization and a feeling of isolation, was more serious than the musculoskeletal pain. Medical intervention was inadequate; poor medical treatment and a lack of patient education exacerbated the feelings of isolation. The termination of the compensation period and the return to work were determined without proper explanation or participation from the injured worker. The return to work process was difficult for injured workers because no work placement rules have been implemented.

A negative interaction between the injured workers and the workers’ compensation insurer has been reported consistently in previous studies [10]. In our study, KCOMWEL’s mistrust and stigmatization contributed to workers’ negative experiences with the workers’ compensation system. Moreover, workers felt the approval rate by IACI is low and the work-relatedness evaluation process is unfair. After introduction of the Occupational Disease Award Commission (ODAC) in 2008, the approval rate was lowered. This fact aggravated workers’ complaints. There were some improvements of approval process in 2012. Those included change of ODAC members composition, elaborating a site inspection and remedy of ‘alteration approval process’ when disease diagnosis was wrong [13]. These changes might affect the approval rates, and follow-up studies are needed.

In particular, the compulsory termination of the compensation period without consideration for the injured worker’s work capacity made the injured workers feel that the compensation period was insufficient and created anxiety about returning to work. There is no limit to the injured worker’s recuperation in many countries, because the purpose of workers’ compensation is not rapid and economically efficient treatment but early and proper treatment to improve the injured worker’s condition and return to work [14]. In Korea, many researchers have proposed a transition in the workers’ compensation appraisal frame from an assessment based on compensation cost or compensation duration to an outcome-based evaluation that includes rehabilitation and return to work. KCOMWEL established the rehabilitation and consult program ‘Personalized Integrated Service’ to arouse the injured workers’ rehabilitation volition and early return to work [15]. However, these legal and administrative improvements do not work in practice. A change in the attitude of the medical staff regarding work-related MSD and quality management for the medical care system as well as administrative changes are necessary to innovate the workers’ compensation system [9].

Workers with musculoskeletal injuries had ambivalent attitudes towards the duration of the compensation period. Many injured workers felt that the duration was short due to the lack of workplace-based rehabilitation, remaining physical symptoms, and a shortage of medical explanations for the disease prognosis. However, injured workers spent ‘one hour per day’ receiving physical therapy, and stayed home alone for the rest of the day during convalescence. For this reason, injured workers regarded convalescence as ‘worse than returning to work with pain.’ This suggests that a confrontation about the workers’ compensation convalescence could be solved by ameliorating the quality of medical treatment and its effects.

We questioned in-depth interview participants of family relationship during convalescence (How was your family relationship and situation during convalescence?), but few statements were corroborated by participants. In questionnaire survey, we also made the same inquiries but most of respondents (82.4%) answered there was no change in family relationship during convalescence. In previous researches, family motivated return to work for feeling obliged to support family living and maintaining family role [16],[17]. Our results might be drawn from a biased group that all of our participants were male workers and they had little financial difficulties during convalescence.

Many study participants experienced more emotional distress than physical pain due to the stigmatization of musculoskeletal patients. Lippel [18] defined stigma as “the co-occurrence of its components—labeling stereotyping, separation, status loss, and discrimination—and further indicate that for stigmatization to occur, power must be exercised”, and emphasized that stigma imposed by the workers’ compensation system prejudiced the injured worker’s mental health. Injured workers compensated by IACI were discriminated because they were stereotyped as lazy, irresponsible, and making easy money [19]. Employees of large companies, like our participants, are often offered supplementary wages by the company in addition to the temporary incapacity benefits. This financial support provided by the company aggravates the stigma of injured workers among co-workers. Stigmatization is a source of chronic stress with consequent negative effects on mental and physical health [20]. Stress is also associated with the constant threat of being stigmatized. A fear of stigmatization constricts social networks and deteriorates quality of life and mental health [21]. In our study, injured workers who feared stigmatization as a malingerer avoided contact with co-workers and restricted social activity.

Workplace interventions that enable injured workers to return to their roles with dignity are important to attenuate these stigmatizations. Assistance by the employers to provide appropriate accommodations and gradual return to work to facilitate the adjustment of the injured worker would help weaken stigmatization [19]. Hepburn et al. [22] reported that the employer’s early response to workplace injury, the compensation process, and the workplace-based return to work brought some favorable influences to the injured workers’ mental health through decreasing stigmatization and increasing organizational justice. Therefore, the low approval rate of work-related MSD by IACI may strengthen the stigmatization and have a negative effect on injured workers’ mental health. These stigmatizations may also interfere with the early diagnosis and treatment of WRMSD. Stigmatization may also prolong the duration of compensation and increase the social cost of WRMSD [23].

This study has some important policy implications. First, higher quality compensated medical care is needed. In our survey, 40.0% of respondents responded that their medical care during the compensation period was unsatisfactory. Participants also reported that their most painful experience as an injured worker was ‘being neglected.’ Therefore, 78.6% of participants obtained extra non-benefit medical care in addition to the compensated medical care. Quality control of primary medical care institutions is urgently needed because these primary clinics are taking charge of treatment for workers with musculoskeletal injuries. At the same time, a rehabilitation promotion policy needs to be established. Rehabilitation treatment is too difficult to be conducted by primary health care institutions. Therefore, public medical centers such as the Workers’ Compensation hospital need to play an important role in rehabilitation treatment. To reduce gaps in the accessibility of high quality medical care institutions across regions, the Workers’ Health Center could become a mainstay for injured workers’ rehabilitation treatment.

Second, mental and psychological care for injured workers has been suggested repeatedly and is still necessary [24]. Among our questionnaires respondents (n = 131), suicide ideation doubled in the convalescence period (10.2%) compared with the current status (5.5%). Because stigmatization as a malingerer and intimidation with the workers’ compensation process itself cause psychological distress for injured workers, all workers, including those injured by serious accidents as well as those with musculoskeletal injuries, should universally be offered mental and psychological care services. Furthermore, workplace intervention to reduce stigmatization and systematic innovation in the workers’ compensation system should be accomplished in parallel to a personal approach for the mental health of injured workers.

This study has some limitations. First, all our study subjects are employees of one large manufacturing company with a strong labor union. Manufacturing industry workers and workers of large-sized enterprises have longer compensation periods [2]. Therefore, injured workers from the non-manufacturing industry and small-sized enterprises may have very different experiences from participants in this study. For example, study participants received supplemental financial support from the company and did not report any economic predicaments. The labor union also was intimately involved in the workers’ compensation system. Therefore, participants admitted that they were approved more easily than other company workers. However, the fact that even our research participants, who were in relatively favorable conditions, experienced the workers’ compensation system as an ‘emotional suffering’ indicates that the workers’ compensation system is an enormously negative experience for injured workers. Nevertheless, it is urgent to carry out further qualitative studies covering diverse workers experiences, such as, workers from small to medium sized enterprise, workers from non-manufacturing industries, and workers from other socio-economic contexts.

Another limitation of this study is the lack of reports from female injured workers. Female workers’ claims for WRMSD and industrial accidents are lower than male workers. In compensation cases, female workers’ convalescence periods are shorter and medical expenses are lower than male workers. Female workers are vulnerable to take information about the workers’ compensation insurance system [25]. Gender as a social position influences the injured workers’ experiences differently. Therefore, future research needs to focus on the experience of female injured workers.

Finally, our study is based on participants’ recall of industrial accident convalescence for MSD over the past 10 years. Many study participants experienced their industrial accident convalescence in 2003. In addition, some participants did not experience KCOMWEL’s recent administrative changes, such as the ‘Personalized Integrated Service’. This administrative change, especially case manager system, is expected to increase the rate of return-to-work and the quality of convalescence [15]. And it is necessary to evaluate and invigorate these services. However, 9 of the 16 in-depth interviewees experienced the second industrial accident convalescence between 2005 and 2013. In addition, 7 of these 9 people were compensated for a newly developed disease different from the disease in the first compensation. And they shared these experiences with us. This fact reduces this limitation of the study.

Despite these potential problems, this study investigates improvement directions for the industrial accident compensation system for MSDs by exploring injured workers’ experiences.

## Conclusions

This study suggests that MSD injured workers found the workers’ compensation system to be intimidating. These injured workers suffered from more emotional distress than physical illness. These workers were treated inadequately and remained isolated for most of the recuperation period. Finally, the compensation period was terminated without ample guidance or an appropriate rehabilitation process. Interventions to alleviate these negative experiences, such as quality control of the medical care institutions and provisions for mental and psychological care for injured workers, are needed to help injured workers return to work earlier and more healthy.

## Abbreviations

IACI: Industrial Accident Compensation Insurance

MSD: Musculoskeletal disease

WRMSD: Work-related musculoskeletal disorders

HIVD: Herniated intervertebral disc

KCOMWEL: Korea Workers’ Compensation and Welfare Service

ODAC: Occupational Disease Award Commission

## Competing interests

The authors declare that they have no competing interests.

## Authors’ contributions

MC and HRK designed the study and the analytic strategy. MC, JWL, HRK conducted the in-depth interviews. HEL and JUK supervised the research concept and design, acquisition of data. JSB analyzed the data and helped conduct the literature review. All authors read and approved the final manuscript.
